# MiR-1303 Regulates Mycobacteria Induced Autophagy by Targeting Atg2B

**DOI:** 10.1371/journal.pone.0146770

**Published:** 2016-01-15

**Authors:** Kin Yi Au, John C. H. Pong, Wai Lim Ling, James C. B. Li

**Affiliations:** Department of Paediatrics and Adolescent Medicine, Li Ka Shing Faculty of Medicine, The University of Hong Kong, Hong Kong, China; Cleveland Clinic, UNITED STATES

## Abstract

MicroRNAs are emerging post-transcriptional regulators of gene expressions in both innate immunity and adaptive immunity. In mycobacteria infection, autophagy plays an important role in innate defense mechanism and is tightly regulated by the autophagy-related proteins. Here, we show that Atg2B is involved in the regulation of mycobacteria-induced autophagy. MiR-1303, which function is not defined yet, is found to negatively regulate mycobacteria-induced Atg2B protein production, ultimately down-regulate mycobacteria-induced autophagy. MiR-1303 production is shown to be upregulated during BCG infection and its production is regulated by PI3K and NFκB. It is also demonstrated that miR-1303 targets putative target sites on Atg2B and possibly represses its translation.

## Introduction

Autophagy was first known to be a highly regulated process for recycling of intracellular protein and organelles. Constitutive autophagy is commonly found in cells to carry out the basal housekeeping role of eliminating damaged organelles. Autophagy can also be upregulated in responses to nutritional, differentiation and danger signals [1, 2]. It therefore also serves essential roles in both innate and adaptive immunity. Dysregulation of autophagy has been implicated in pathogenesis of a broad spectrum of diseases including neural diseases, metabolism defects, cancers and infectious disease [2, 3]. During *Mycobacterium tuberculosis* infection, autophagy acts as an innate immune defense mechanism and modulates inflammatory responses. And the progression of autophagy is tightly regulated by a family of autophagy regulators, autophagy-related proteins (Atg) [4, 5]. Atgs control the core pathways of autophagy including induction, vesicle nucleation, vesicle elongation, retrieval and fusion [6]. Atg2 is known to be involved in the retrieval process of autophagy in yeast. Atg2 and Atg18 bind to Atg9 and participate in the trafficking of Atg9 [7, 8]. The retrieval process involves shuttling of the transmembrane protein Atg9 between endosomes and the trans-Golgi network [8]. It is suggested that the trafficking of Atg9 recruits lipids and regulatory protein to the growing phagophore and potentially contributes to the delivery of membrane to the pre-autophagosomal structure (PAS) [6]. Mammalian Atg2 homologs, Atg2A and Atg2B have also been shown to participate in autophagy in human cells. Recent studies indicate that dysregulation of microRNAs can contribute to the defect in autophagy and autophagy-related disease [9, 10].

MicroRNAs (miRNAs) comprise a family of single stranded, non-coding, short (20–23 bp) RNAs that act as post-transcriptional regulators of gene expressions. The first observation of miRNA regulatory effect was in *Caenorhabditis elegans* in 1993 [11]. During past two decades, miRNAs have emerged as important regulators in eukaryotic organisms. MiRNAs are predicted to regulate activity of ~50% of all protein-encoding genes in mammals [12]. MiRNAs genes are transcribed by RNA polymerase II to generate the precursor molecules, primary transcript (pri-miRNAs) in nucleus. The pri-miRNAs fold into hairpins, which are then processed subsequently by two members of RNase III enzyme family, Drosha and Dicer. One strand of the hairpin duplex is incorporated into effector complex Argonaute family protein (AGO) to form the core of miRNA-induced silencing complexes (miRISCs). miRISCs silence the expression of target genes at the post-transcriptional level [12–15]. MiRNA-mediated regulations were shown to be essential in many developmental and cellular processes, such as innate immunity, metastasis and cellular metabolism [13]. Since the first publication uncovered the regulation of BECN1 by miR-30A [16], emerging reports show that miRNAs target the Atgs and associate with cancers [6, 9]. However, the roles of miRNAs in autophagy during infection remain not fully illustrated. We herein show that miR-1303, a miRNA which function is not yet defined, targets Atg2B and ultimately regulates mycobacteria-induced autophagy.

## Materials and Methods

### Reagents

The specific chemical inhibitors for PI3K (LY 294002), ERK 1/2 (U0216), p38 (SB203580) and NF-κB translocation was purchased from Calbiochem, USA and diluted in DMSO. Antibody against LC3B was purchased from Cell Signaling Technology, USA and antibody against Atg2B was purchased from Abcam, UK. Antibody against Actin was purchased from Santa Cruz Biotechnology, USA. Goat anti-rabbit IgG HRP-conjugated secondary antibody was purchased from BD Bioscience, USA and peroxidase-conjugated rabbit anti-goat IgG antibody was from Dako, Denmark.

### Cell cultures

Human primary blood monocyte derived macrophages (PBMacs) were isolated from the buffy coats of healthy blood donors (Hong Kong Red Cross Blood Transfusion Service) by Ficoll-Paque(GE Healthcare Medical Systems, USA) centrifugation and purified by using an adherence method. Monocytes were seeded onto tissue culture plates and differentiated in RPMI 1640 medium (Invitrogen, USA) supplemented with 5% heat-inactivated autologous plasma. Differentiated macrophages were obtained after 14 days of culture in vitro and were seeded in quantities of 5×10^5^ cells per well into 24-well tissue culture plates [17]. All manipulations were performed in compliance with the approval by the Institutional Review Board of the University of Hong Kong/Hospital Authority Hong Kong West Cluster. HEK293T/17 (ATCC, Cat# CRL-11268, USA) was directly purchased from ATCC, USA. The cell line was cultured and seeded in quantities of 5×10^4^ cells per 48-well tissue culture plates in DMEM (Invitrogen, USA) supplemented with 10% fetal calf serum (Life technology, USA) and 1% penicillin/streptomycin (Life technology, USA).

### Mycobacteria

Bacillus Calmette Guerin (BCG vaccine, Danish strain 1331) (Statens Serum Inititut, Danish) was reconstituted in diluent by the manufacturer.

Green fluorescent protein labeled Bacillus Calmette Guerin (GFP-BCG) was kindly provided by Professor Yossef Av-Gay of the University of British Columbia, Canada and quantified on 7H10 agar plates (BD Bioscience, USA). The mycobacteria were resuspended in PBS with 10% glycerol and stored at -70°C.

### Transfection

Negative control siRNAs or Atg2B siRNA (200 nM) (Invitrogen, USA) were transfected into cells using LipofactamineTM2000 (Invitrogen, USA) according to the manufacturer’s protocol for 48 hrs before indicated treatments. Negative control mimics [Ambion Pre-miRTM Negative control #1] or miR-1303 mimics [Ambion Pre-miRTM miRNA Precursor] (Ambion, USA) were transfected into cells using LipofactamineTM2000 (Invitrogen, USA) according to the manufacturer’s protocol for 24 hrs before indicated treatments.

### Quantitative reverse transcription polymerase chain reaction (qRT-PCR)

Total RNA was extracted by TRIzol Reagent (Invitrogen, USA). RNAs was then reverse-transcribed into cDNAs by using oligo[dT] (Genome Research Center, HKU, PRC) and SuperScript II System (Invitrogen, USA) following the manufacturer’s protocol. Quantitative PCR was performed by using TaqMan gene-specific assays-on-demand reagent kits (Applied Biosystems, USA).cDNA was amplified with the kit reagents and gene-specific probes in the ABI Prism 7500 Sequence Detection System (Applied Biosystems, USA). By using the comparative cycle number to threshold (Cτ) method, the mRNAs levels of Atg2B were normalized to the mRNA level of a reference gene GAPDHs. Quantitative PCR of miR-1303 was done using Taqman MicroRNA assay kit (Applied Biosystems, USA). MiRNAs levels were normalized to RNU48.

### Western blot analysis

Total cellular lysates were obtained by using lysis buffer containing 50 mM Tris—HCl (pH7.4), 50 mM NaCl, 0.1mM EDTA, 50 mM sodiumfluoride (NaF), 10 mM beta-glycerophosphate, 10% glycerol, 1% Triton X-100, 1 M phenylmethanesulfonylfluoride(PMSF), 2mg/ml aprotinin, 1 mM sodium orthovanadate, 2mg/ml leupeptin and 2mg/ml pepstatin. The homogenate was collected and the protein concentrations determined with BCA protein assay reagent kit (ThermoFisher Scientific, USA). Protein samples were mixed with sample buffer containing 100nM Tris-HCL (pH 6.8), 200 mM DTT, 4% SDS, 0.2% bromophenol blue and 20% glycerol, followed by boiling for 10 mins. The protein components were separated by either 7.5% or 13% SDS—PAGE and electroblotted onto nitrocellulose membranes (Schleicher& Schuell, Germany), followed by probing overnight with specific antibodies for Atg2B or LC3B or actin. After washing with 1% Tris-buffered saline with Tween 20 (TBST), the membranes were incubated with the corresponding secondary antibodies. The signals were visualized with an Enhanced Chemiluminescence System (GE Healthcare, USA).

### Luciferase assay

Oligonucleotides with miR-1303 putative target sites or mutated target sites on Atg2B were annealed and cloned into the XbaI site of the pGL3 luciferase control vector (Promega, USA). HEK293T/17 cells were transfected with 50 ng of firefly luciferase reporter vector and 5 ng of pRL-TK Renilla luciferase control vector (Promega, USA) using LipofactamineTM2000 (Invitrogen, USA) for 4 hrs, followed by transfection of miR-1303 mimics. Assays were performed 14 hrs after transfection using Dual-Luciferase Reporter assay system (Promega, USA). Firefly luciferase activity was normalized to Renilla luciferase activity.

### Immunocytochemistry

PBMacs were fixed in 4% paraformaldehyde and permeabilized with 0.25% Triton X-100. Following incubation with 1% BSA in PBS for blocking, fixed cells were stained with specific antibody for LC3B. Nuclei of cells were stained with 4,6-diamidine-2-phenylindole dihydrochloride (DAPI). Fluorescence images were taken by a Axioplan 2 imaging system (META; Carl Zeiss Inc., Germany).

### Statistical analysis

The data were analyzed by one-way ANOVA by using GraphPad Prism. P values < 0.05 were considered as statistically significant.

## Results

### BCG induces miR-1303 production

**Fig 1:**
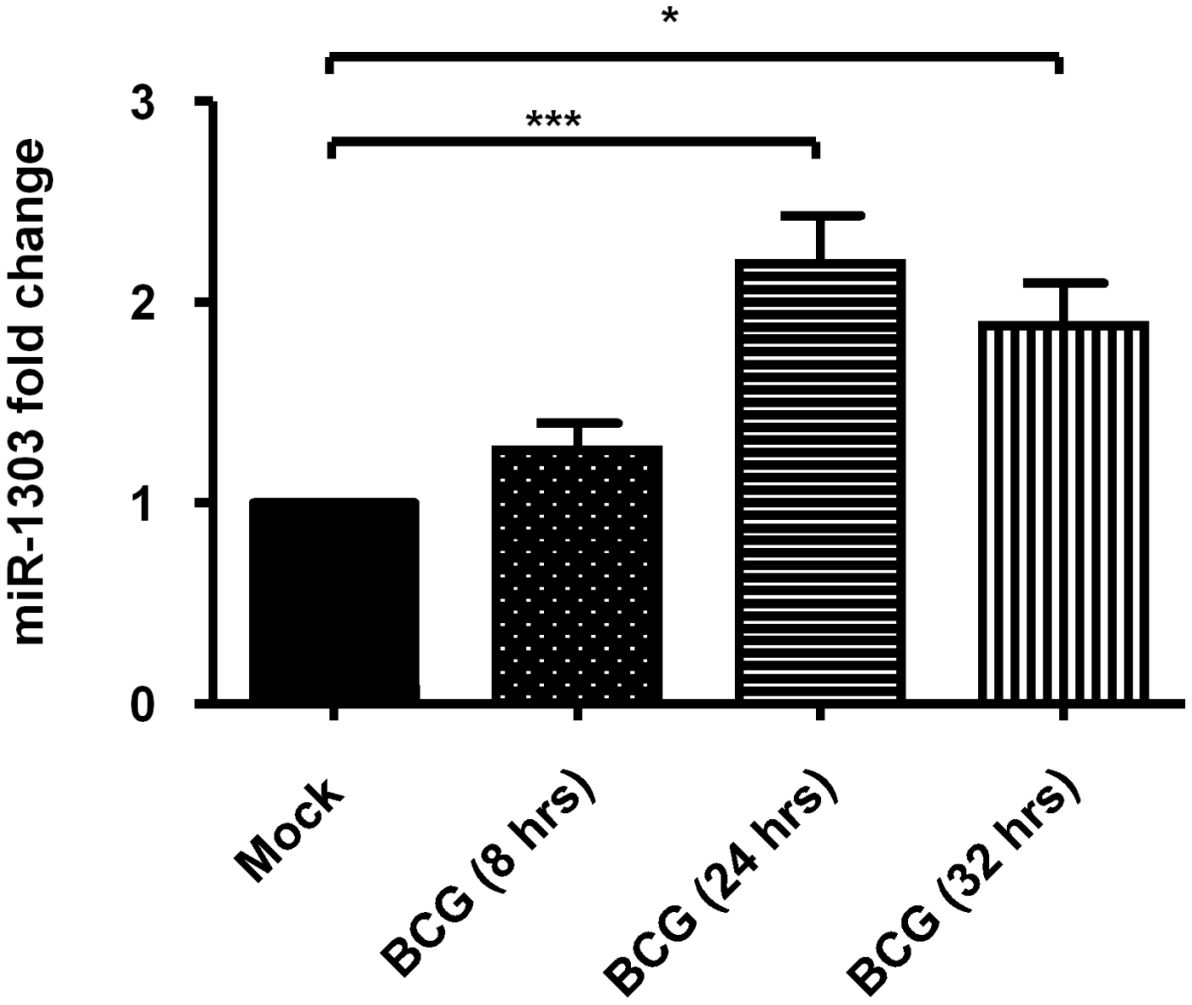
BCG induces miR-1303 production. PBMacs were treated with Mock (diluent for BCG) for 8 hrs, 24 hrs and 32 hrs respectively and BCG (MOI = 1) for indicated intervals. Total RNA was extracted and reverse-transcribed into cDNA. The miR-1303 levels normalized to that of RNU48 were analyzed by quantitative PCR using the cDNA. The miR-1303 levels in cells treated with BCG were expressed as fold change relative to those in mock-treated cells respectively. The data are expressed as the mean ±SEM of independent experiments using PBMacs from five individuals. *, p<0.05, ***, p<0.001

MiRNAs have been shown to play important roles in innate immunity. To identify the miRNAs which might be involved in innate immunity during BCG infection, PBMacs were infected with BCG (MOI = 1) for 8 hrs or 24 hrs and the level of RNAs were compared on a MicroRNA qPCR array. The results showed that miRNAs (i.e. miR-146a, miR-155, let-7 family etc.) which are well known to regulate innate immunity were upregulated during BCG infection [data not shown]. Of note, miRNAs (i.e. miR-1303) which functions are not known were upregulated during BCG infection. The level of BCG-induced miR-1303 was further validated by QPCR. It was found that BCG (MOI = 1) upregulated miR-1303 level by 2.2 fold and 1.9 fold upon 24 hrs and 32 hrs of BCG infection respectively (Fig 1).

### BCG-induced miR-1303 production is regulated by PI3K and NF-κB

**Fig 2:**
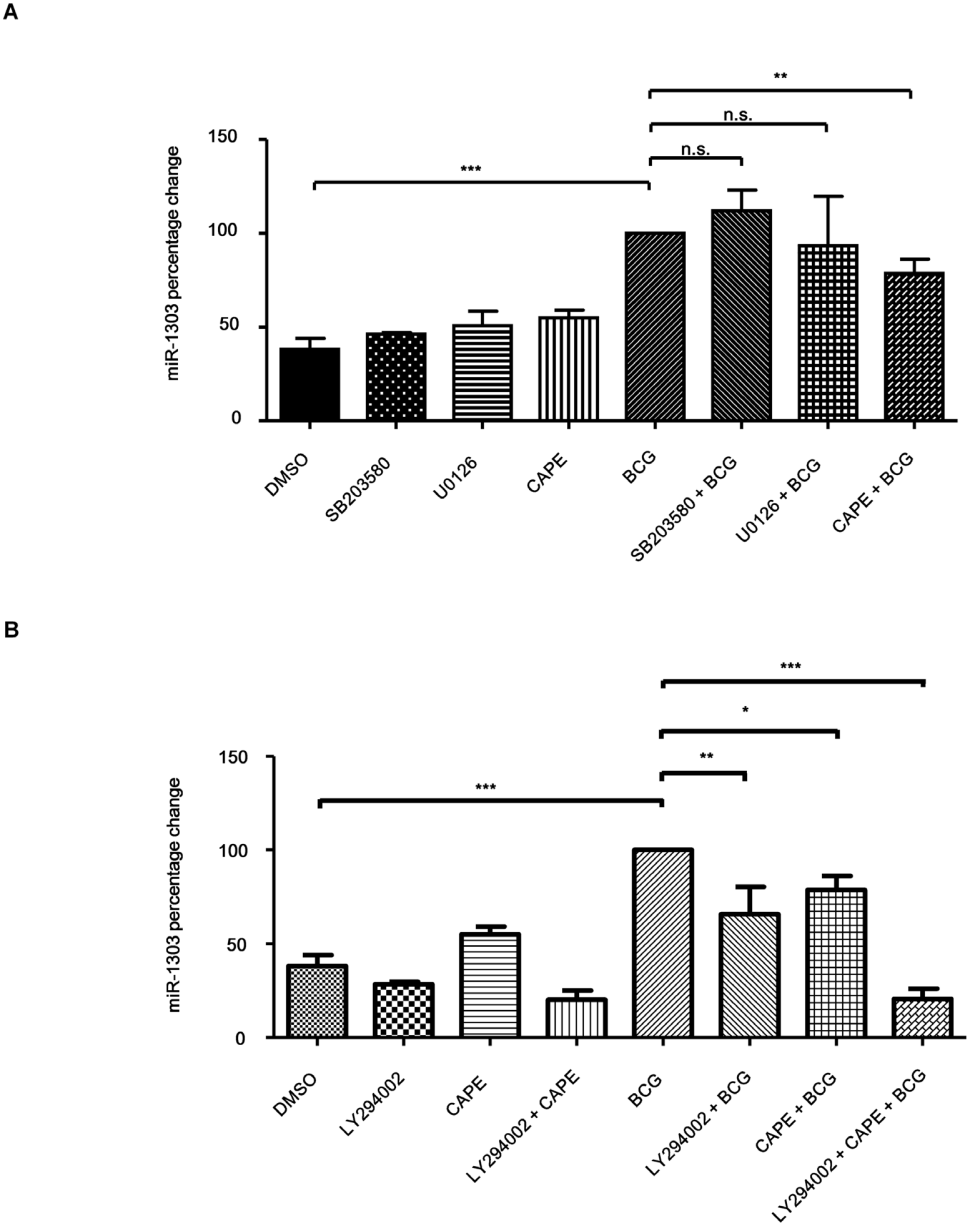
BCG-induced miR-1303 production is regulated by PI3K and NF-κB. **(A)**PBMacs were treated with DMSO (0.05%) or SB203580 (10 μM) or U0216 (10μM) or CAPE (15 μg/ml), followed by Mock or BCG (MOI = 1) for 24 hrs. Total RNA was extracted and reverse-transcribed into cDNA. The miR-1303 levels normalized to that of RNU48 were analyzed by quantitative PCR using the cDNA. The miR-1303 levels in cells were expressed as percentage relative to those in DMSO-BCG-treated cells. The data are expressed as the mean ±SEM of independent experiments using PBMacs from six individuals. n.s., not significant. **, p<0.01. ***, p<0.001. (B) PBMacs were treated with DMSO (0.05%) or LY294002 (10 μM) or CAPE (15 μg/ml) or LY294002 (10 μM) + CAPE (15 μg/ml), followed by Mock or BCG (MOI = 1) for 24 hrs. Total RNA was extracted and reverse-transcribed into cDNA. The miR-1303 levels normalized to that of RNU48 were analyzed by quantitative PCR using the cDNA. The miR-1303 levels in cells were expressed as percentage relative to those in DMSO-BCG-treated cells. The data are expressed as the mean ±SEM of independent experiments using PBMacs from five individuals. *,p<0.05, **, p<0.01. ***, p<0.001.

Then we further delineated the regulation of BCG-induced miR-1303 level. PBMacs were first treated with ERK1/2 inhibitor (U0126, 10 μM) or p38 MAPK kinase inhibitor (SB203580, 10 μM) or NF-κB p65 subunit translocation inhibitor (Caffeic acid phenethyl ester, CAPE, 15 μg/ml), then BCG (MOI = 1) were added to infect the cells. It was shown that CAPE inhibited BCG-induced miR-1303 level by 21.3% while other inhibitors did not affect the BCG-induced miR-1303 level (Fig 2A). In addition, we further examined the regulation by using PI3K (phosphoinositide 3-kinase) inhibitor, LY294002 (10 μM). PBMacs were first treated with LY294002 (10 μM) or with LY 294002 (10 μM) and CAPE (15 μg/ml), then BCG (MOI = 1) were added to infect the cells. It was shown that LY294002 inhibited BCG-induced miR-1303 level by 34.14% (Fig 2B).With pretreatment of combination of two inhibitors, BCG-induced miR-1303 level was suppressed synergistically by 79.5% (Fig 2B). Together the results show that BCG-induced miR-1303 production is regulated by both NFκB and PI3K.

### BCG induces autophagy which involves Atg2B

**Fig 3:**
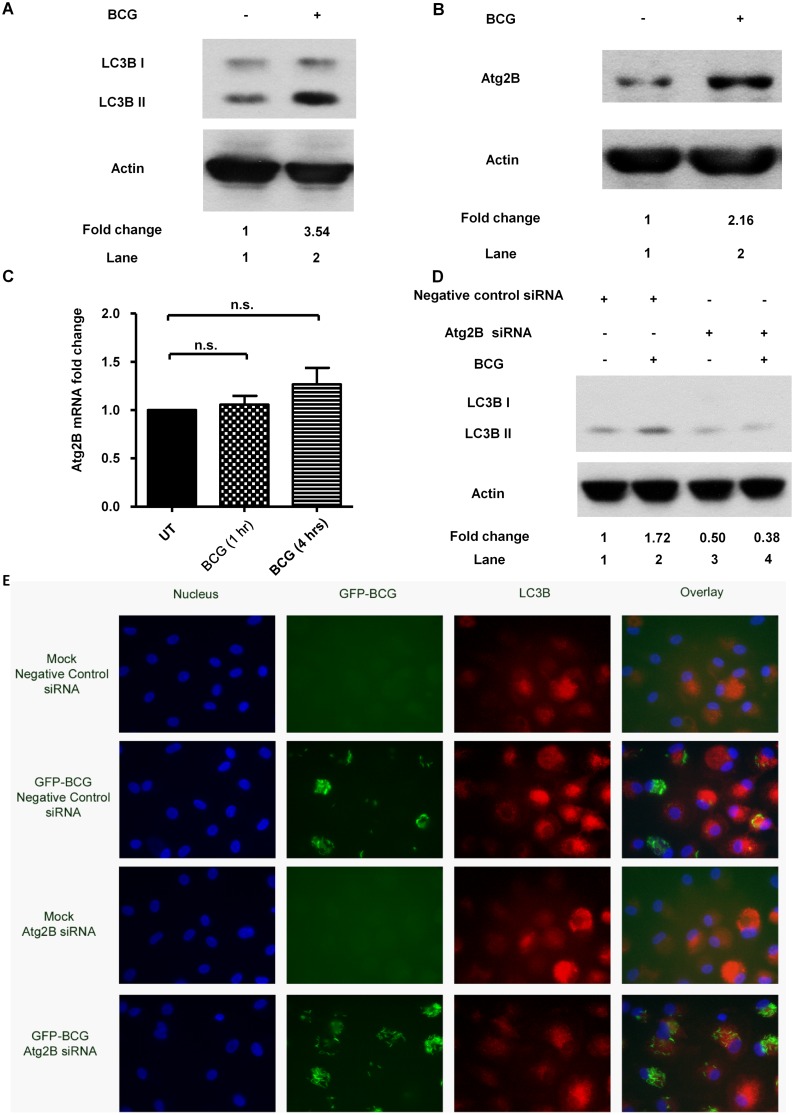
BCG induces autophagy which involves ATG2B. **(A)** PBMacs were treated with BCG (MOI = 1) for 24 hrs. Cell lysates were examined by Western blot using anti-LC3B or anti-actin antibodies, as indicated. The results shown are representative of independent experiments using PBMac from four individuals. The intensity of the relevant bands was quantified by laser densitometry. The ratio of intensities of LC3B-II/actin in each sample was compared with that in cells treated with Mock. **(B)** PBMacs were treated with BCG (MOI = 1) for 24 hrs. Cell lysates were examined by Western blot using anti-Atg2B or anti-actin antibodies, as indicated. The results shown are representative of independent experiments using PBMacs from three individuals.The intensity of the relevant bands was quantified by laser densitometry. The ratio of intensities of Atg2B/actin in each sample was compared with that in cells treated with Mock. **(C)** PBMacs were treated with Mock (diluent for BCG) for 1 hrs and 4 hrs respectively and BCG (MOI = 1) for indicated intervals. The Atg2B mRNA levels normalized to that of GAPDH were analyzed by quantitative PCR. The Atg2B mRNA levels in cells were expressed as fold change to those in Mock-treated cells. The data are expressed as the mean ±SEM of independent experiments using PBMacs from seven individuals. n.s., not significant. **(D)** PBMacs were transfected with Atg2B siRNA (200 nM) or negative control siRNA (200 nM) for 48 hrs, followed by BCG (MOI = 1) treatment for 24 hrs. Cell lysates were examined by Western blot using anti-LC3B or anti-actin antibodies, as indicated. The results shown are representative of independent experiments using PBMac from four individuals. The ratio of intensities of LC3B-II/actin in each sample was compared with that in cells treated with Mock and negative control siRNA. **(E)** PBMacs were transfected with Atg2B siRNA (200 nM) or negative control siRNA (200 nM) for 48 hrs, followed by GFP-BCG (MOI = 1) treatment for 24 hrs. PBMacs were then fixed and stained with anti-LC3B antibodies and DAPI. Images were taken with a Carl Zeiss fluorescence microscope system in independent experiments using PBMacs from four individuals. Representative images from five fields per well are shown.

Before we delineated the role of miR-1303, the pathways involved in BCG-induced autophagy were investigated. It was shown that BCG induced LC3B II protein expression (Fig 3A) and Atg2B protein expression (Fig 3B) while did not affect the Atg2B mRNA level (Fig 3C). It was then further investigated if BCG induced autophagy which involved Atg2B protein expression. With knockdown of Atg2B by Atg2B siRNA (200 nM) transfection for 48 hrs, BCG-induced autophagy was suppressed (Fig 3D). The results were further validated by observation under fluorescence microscope. PBMacs were first transfected with Atg2B siRNA (200 nM) for 48 hrs, then infected with GFP-BCG for 24 hrs. It was shown that knockdown of Atg2B suppressed the GFP-BCG-induced LC3B-positive autophagosomes formation (Fig 3E).

### miR-1303 regulates autophagy related gene Atg2B expression

**Fig 4:**
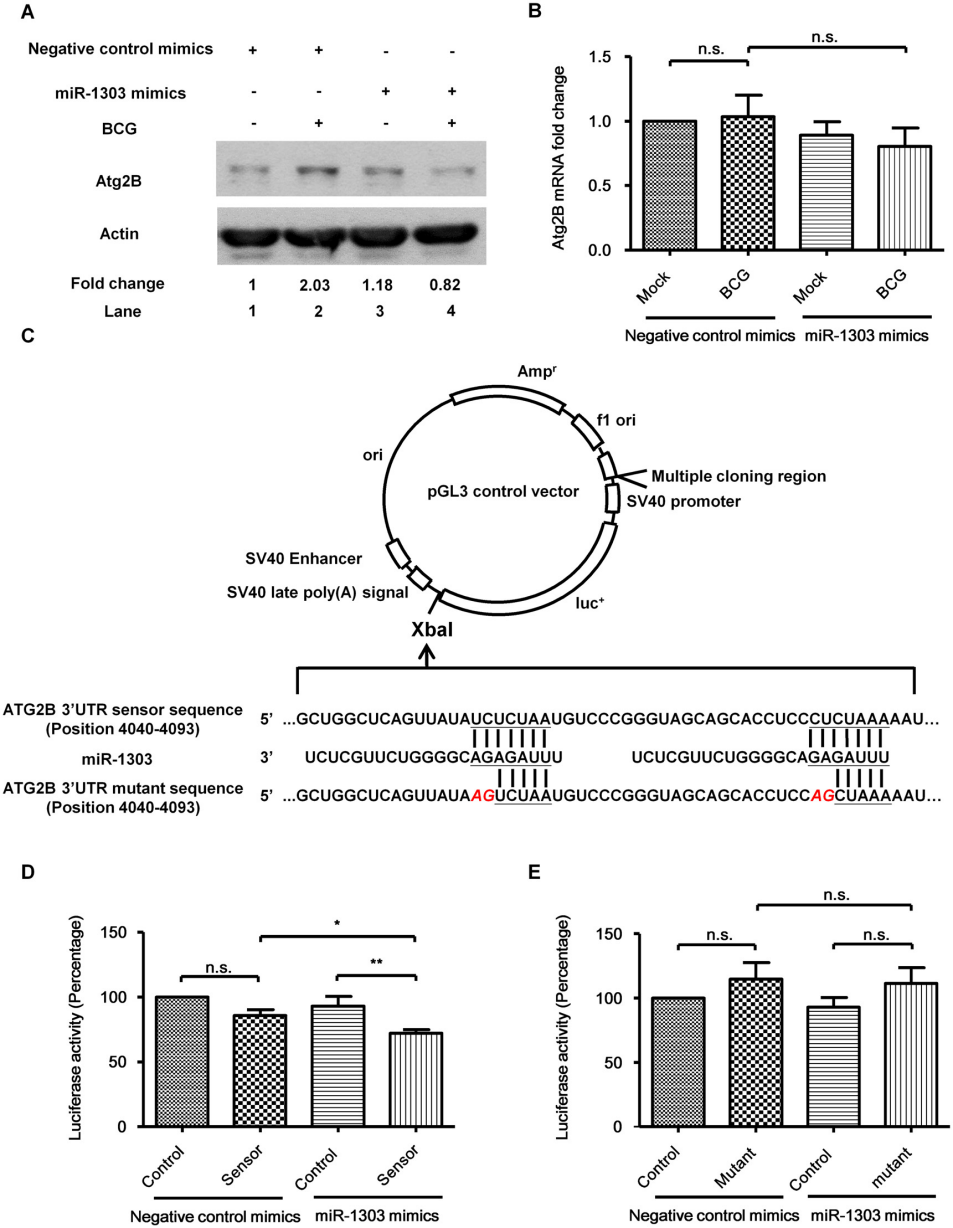
miR-1303 regulates autophagy related gene Atg2B expression. **(A)** PBMacs were transfected with miR-1303 mimics (40 nM) or negative control mimics (40 nM) for 24 hrs, followed by BCG (MOI = 1) treatment for 24 hrs. Cell lysates were examined by Western blot using anti-Atg2B or anti-actin antibodies, as indicated. The results shown are representative of independent experiments using PBMac from four individuals. The ratio of Atg2B/actin in each sample was compared with that in cells treated with Mock and negative control mimics. **(B)** PBMacs were transfected with miR-1303 mimics (40 nM) or negative control mimics (40 nM) for 24 hrs, followed by BCG (MOI = 1) treatment for 1 hr. Total RNA was extracted and reverse-transcribed into cDNA. The Atg2B mRNA levels normalized to that of GAPDH were analyzed by quantitative PCR using the cDNA. The Atg2B mRNA levels in cells were expressed as fold change to those in Mock-negative control mimics-treated cells. The data are expressed as the mean ±SEM of independent experiments using PBMacs from eight individuals. n.s., not significant. **(C)** Schematic representations of the miR-1303 putative target sites (sensor) and mutated target sites (mutant) of the 3’UTR of Atg2B which were cloned into the pGL-3 luciferase control construct. **(D)** HEK293T/17 cells were transfected with empty pGL3 luciferase control vector (control) or pGL3 luciferase control vector with miR-1303 putative target sites on Atg2B (sensor) for 4 hrs and then transfected with negative control mimics or miR-1303 mimics (40 nM) for 14 hrs. pRL-TK renilla luciferase reporter vector were transfected as transfection control. Luciferase activities of cell lysates were assayed using Dual-Luciferase Reporter assay System. The luciferase activity ratio of pGL3/pRL-TK in each sample was compared with that in cells transfected with control and negative control mimics. The data are expressed as the mean±SEM of six independent experiments. n.s., not significant. *, p<0.05. **, p<0.01. **(E)** HEK293T/17 cells were transfected with empty pGL3 luciferase control vector (control) or pGL3 luciferase control vector with miR-1303 mutated target sites on Atg2B (mutant) for 4 hrs and then transfected with negative control mimics or miR-1303 mimics (40 nM) for 14 hrs. pRL-TK renilla luciferase reporter vector were transfected as transfection control. Luciferase activities of cell lysates were assayed using Dual-Luciferase Reporter assay System. The luciferase activity ratio of pGL3/pRL-TK in each sample was compared with that in cells transfected with control and negative control mimics. The data are expressed as the mean±SEM of four independent experiments. n.s., not significant.

Next we investigated if miR-1303 regulated the BCG-induced autophagy. MiR-1303 is a miRNA with 22 nucleotides, first identified and sequenced from human embryonic stem cells and embryoid body samples [18]. However, its function is unknown. Therefore, in silico search for putative binding targets of miR-1303 was done by employing online computational algorithm. Both TargetScan (http://www.targetscan.org) and miRDB (http://mirdb.org/miRDB/) indicate putative target sites of miR-1303 in 3’UTR of Atg2B. To verify if the miR-1303 regulated the Atg2B, miR-1303 was over expressed to see the effect on Atg2B expression. PBMacs were first transfected with miR-1303 mimics (40 nM) for 24 hrs to overexpress miR-1303, then BCG (MOI = 1) were added to infect the cells for 24 hrs. It was shown that overexpression of miR-1303 significantly suppressed BCG-induced Atg2B protein production (Fig 4A), although miR-1303 overexpression did not affect the Atg2B mRNA level (Fig 4B). Furthermore, to confirm the direct interaction between miR-1303 and its binding site, HEK293T/17 cells were transfected with pGL3 luciferase control construct (control) or pGL3 luciferase construct with putative target sites on Atg2B (sensor) (Fig 4C). It was demonstrated that the luciferase activity was slightly decreased when putative binding site was inserted in the construct (Fig 4D). This might due to the binding of constitutive production miR-1303 in HEK293T/17 (data not shown). And with overexpression of miR-1303, the luciferase activity was further decreased (Fig 4D). Importantly, the decrease of luciferase activity was abolished when the target sites were mutated. When HEK293T/17 cells were transfected with pGL3 luciferase construct with mutated putative target sites on Atg2B (mutant) for 4 hrs and then transfected with miR-1303 mimics (40 nM) for 14 hrs, the miR-1303 could not bind to the mutated construct and failed to repress the luciferase activity (Fig 4E). Together the results indicate that miR-1303 binds to putative target sites on Atg2B and represses the translation of Atg2B. However the process is independent of mRNA decay.

### miR-1303 regulates autophagy

**Fig 5:**
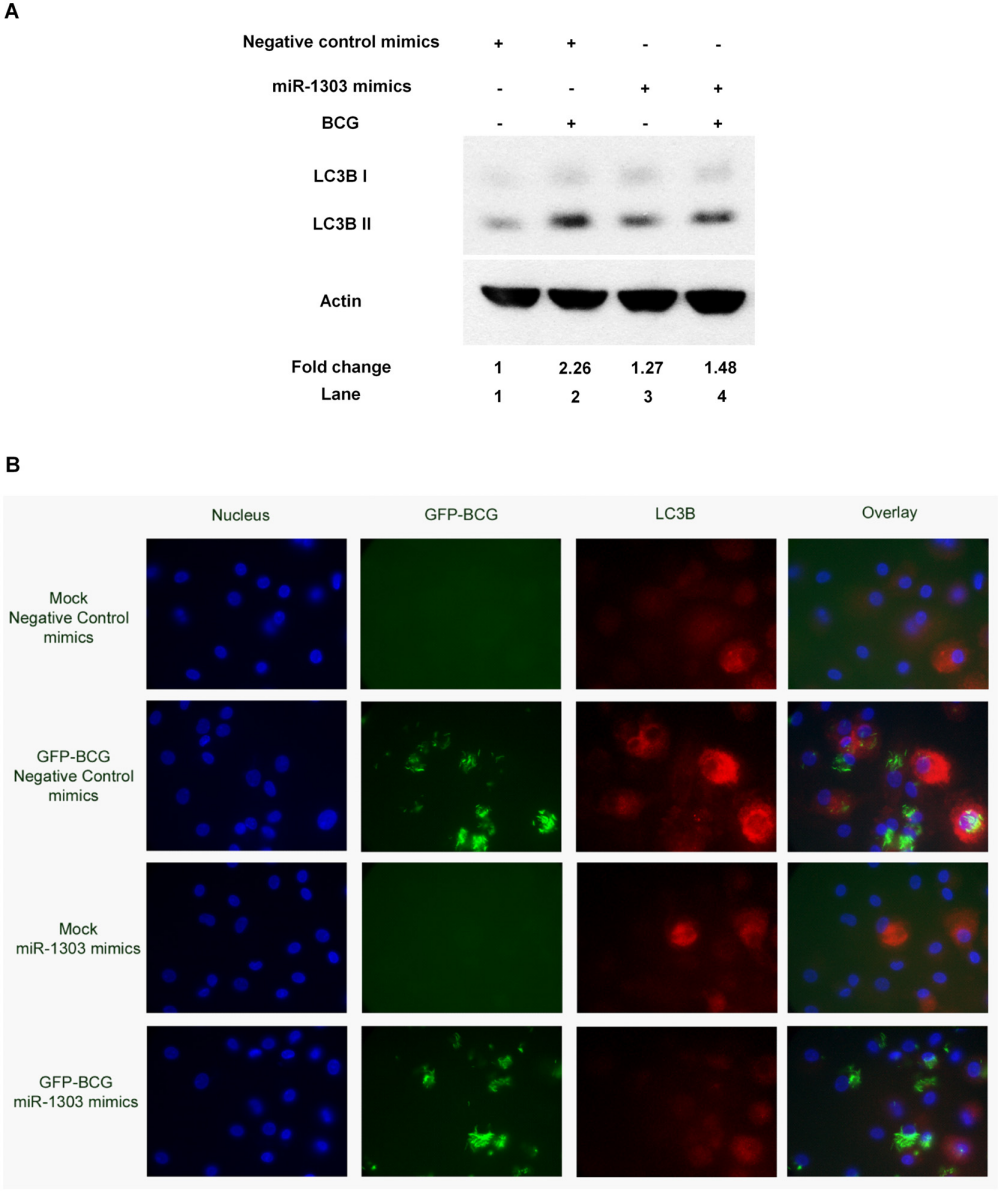
miR-1303 regulates autophagy. **(A)** PBMacs were transfected with miR-1303 mimics (40 nM) or negative control mimics (40 nM) for 24 hrs, followed by BCG (MOI = 1) treatment for 24 hrs. Cell lysates were examined by Western blot using anti-LC3B or anti-actin antibodies, as indicated. The results shown are representative of independent experiments using PBMac from four individuals. The ratio of LC3B-II/actin in each sample was compared with that in cells treated with Mock and negative control mimics. The data are expressed as the mean±SEM of independent experiments using PBMacs from four individuals. **, p<0.01. **(B)** PBMacs were transfected with miR-1303 mimics (40 nM) or negative control mimics (40 nM) for 24 hrs, followed by GFP-BCG (MOI = 1) treatment for 24 hrs. PBMacs were then fixed and stained with anti-LC3B antibodies and DAPI. Images were taken with a Carl Zeiss fluorescence microscope system in independent experiments using PBMacs from five individuals. Representative images from five fields per well are shown.

Furthermore, we investigated if the miR-1303 suppressed Atg2B protein, ultimately downregulated autophagy. PBMacs were first transfected with miR-1303 mimics (40 nM) for 24 hrs to overexpress miR-1303, then BCG (MOI = 1) were added to infect the cells for 24 hrs. It was demonstrated that the LC3B II expression was decreased with the overexpression of miR-1303 (Fig 5A). The results were further validated by observation under fluorescence microscope. PBMacs were first transfected with miR-1303 mimics (40 nM) for 24 hrs, then infected with GFP-BCG for 24 hrs. It was shown that overexpression of miR-1303 suppressed the GFP-BCG-induced LC3B-positive autophagosomes formation (Fig 5B). Herein, we report that miR-1303 suppresses Atg2B protein production and leads to downregulation of autophagy.

## Discussion

MiRNAs comprise a family of single stranded, non-coding, short RNAs that act as post-transcriptional regulators of gene expressions. MiRNA-mediated regulations have been shown to be essential in many developmental and cellular processes [13]. Emerging studies have characterized the changing profiles of host miRNAs during infection [19]. Important roles of miRNAs in regulation of innate and adaptive immune responses have been revealed [20]. MiRNAs can regulate immune responses from receptor to cell signaling molecules and ultimately to the cytokine expression. In our study, differential expressions of miRNAs have been found during mycobacteria infection (date not shown). MiR-146a, which regulates functions in innate immune cells upon LPS stimulation [21, 22], was also induced upon mycobacteria infection (S1 Fig). Consistent with publications from other groups [21–23], miR-146 was found to regulate mycobacteria-induced IL-1β production in human primary macrophage (S2 Fig). In addition to the known miRNAs, miR-1303, miRNA with unknown function was drawn to attention. Herein, we show that miR-1303 is a regulator for autophagy.

Autophagy is a tightly regulated process for recycling intracellular protein and eliminating damaged organelles [1]. Starvation has been well-studied to induce autophagy and harness mycobacterial infection. Mycobacteria itself can also induce autophagy in the absence of starvation or rapamycin. C57BL/6 mice bone marrow derived macrophages and RAW 264.7 cells showed induction of autophagy upon mycobacteria infection [24]. In aggreement with the results in mouse macrophage model in the publication, human primary monocyte derived macrophages were reported here to induce autophagy upon mycobacteria infection. Upon induction of autophagy, a family of autophagy regulators autophagy-related proteins (Atg) orchestrates to control the progression of autophagic pathway [4, 5]. Although mycobacteria-induced autophagy was demonstrated to be independent of mTOR [24], the Atgs involved are not well-illustrated. Herein, our results show that the induction of autophagy involves Atg2B. Atg2 is a new comer discovered to participate in autophagic pathways. Atg2B interacts with Atg18 and participates in the bi-directional trafficking of Atg9 in yeast [7]. In mammalian cells, human Atg2 homologues Atg2A and Atg2B are found to be essential for autophagosome formation. Silencing both homologs completely abrogated the increase in LC3-II induced by starvation and lysosomal inhibition [25]. Of note, starvation did not induce Atg2B protein production and the autophagy process was not affected by Atg2B silencing alone. Silencing Atg2B did not affect the LC3-II upon starvation [25]. In our study, BCG induces Atg2B protein production and silencing Atg2B remarkably suppresses the mycobacteria-induced autophagy. This provides further evidence of complexity of autophagy. Differential induction of Atg2B protein production may therefore contribute to diverse regulations of autophagy upon infection and metabolic stress.

We further delineated the regulation of Atg2B protein production upon mycobacteria induction. Propelling evidences indicate that miRNAs are differentially expressed in human cancers and have roles in regulation of the autophagy process. The miRNAs target on the Atg and impact the core autophagy pathway [6]. In chronic lymphocytic leukemia cell line, miR-130a was reported to target on Atg2B [26]. We hereby show that Atg2B can be targeted by a novel miRNA, miR-1303, in human primary macrophages. With overexpression of miR-1303, the luciferase activity of construct with putative binding sites on Atg2B is decreased while the luciferase activity of construct with mutated binding sites does not show the decrease. The binding of the miR-1303 to the target sites leads to the downregulation of mycobacteria-induced Atg2B protein. The common theme emerging from the studies of target regulation by miRNAs is that mRNA degradation provides the main contribution to the reduction of protein level [27]. However, some miRNAs repress their targets through translational repression without detectable change in mRNA levels [28–30]. It is suggested that miRNA can suppress mRNA through initiation of translation by interfering the binding of eIF4E to the cap structure of the mRNA [30]. In our study,BCG significantly induces Atg2B protein expression (Fig 3B). However BCG does not induce Atg2B mRNA production (Fig 3C). BCG possibly acts on the posttranscriptional stage of Atg2B production and increases the translation of Atg2B mRNA. MiR-1303 targets on Atg2B and downregulates the protein level of Atg2B without affecting the mRNA level of Atg2B. The regulation may therefore due to the translational repression instead of destablilization of mRNA. The miRNAs may act on the newly synthesized mRNAs and fine tune the Atg2B protein expression induced upon mycobacteria infection. Further studies of mechanism of translational repression can be made to understand how miR-1303 suppresses the protein level of Atg2B without changing the level of its mRNA.

We then further investigate the down stream effect of Atg2B on the mycobacteria-induced autophagy. It is reported hereby that the suppression of Atg2B ultimately downregulates autophagy. With overexpression of miR-1303, mycobacteria-induced autophagy is suppressed. This result further supports the study in human chronic lymphocytic leukemia that miRNA targets on Atg2B and inhibits autophagy [26].

During mycobacteria infection, autophagy plays a role in the killing of mycobacteria [31]. However, overexpression of miR-1303 does not significantly affect the bacterial load of macrophage (S3 Fig). Interestingly, overexpression miR-1303 reduces TNF-α production (S4 Fig). TNF-α is required for the control of *Mycobacterium tuberculosis* infection. It participates in activation of macrophages and granuloma formation. Autophagy is one of the known regulation processes that controls TNF-α production in PBMC [32]. With overexpression of miR-1303, TNF-α production is shown to be decreased (S4 Fig). This may be due to the repression of mycobacteria-induced autophagy. Excessive TNF-α production can cause pathological effects in *Mycobacterium tuberculosis* infection. Complete absence of TNF can lead to an uncontrolled Th1 cytokine response [33]. Therapeutic application of miR-1303, with inhibitory effects on TNF-α production, may open a new way of controlling TNF-α production.

Taken together, we show that Atg2B participates in the mycobacteria-induced autophagy process. MiR-1303, a novel miRNA, targets on Atg2B and suppresses its expression. Suppression of Atg2B expression ultimately leads to inhibition of autophagy. The study therefore provides an evidence of Atg2B involvement in mycobacteria-induced autophagy and the insight of miRNA regulation of autophagy.

## Supporting Information

S1 Fig BCG induces miR-146a production. PBMacs were treated with Mock (diluent for BCG) for 4 hrs and 24 hrs respectively and BCG (MOI = 1) for indicated intervals. Total RNA was extracted and reverse-transcribed into cDNA. The miR-146a levels normalized to that of RNU48 were analyzed by quantitative PCR using the cDNA. The miR-146a levels in cells treated with BCG were expressed as fold change relative to those in mock-treated cells respectively. The data are expressed as the mean ±SEM of independent experiments using PBMacs from three individuals. **, p<0.01.
(PDF)

S2 Fig miR-146a regulates BCG-induced IL-1β production. PBMac were transfected with Anti-miR Negative control inhibitors (40 nM) or Anti-miR-146a inhibitors (40 nM) for 24 hrs, followed by BCG (MOI = 1) treatment for 24 hrs. Cell supernatants were collected and the cytokine levels in the supernatants were determined by ELISA. The cytokine levels in cells were expressed as percentage relative to those in Anti-miR Negative control inhibitor-BCG (24 hrs)-treated cells. The data are expressed as the mean ±SEM of independent experiments using PBMac from seven individuals. **, p<0.01. ***, p<0.001.
(PDF)

S3 Fig miR-1303 does to affect bacterial load in macrophage. PBMacs were transfected with miR-1303 mimics (40 nM) or negative control mimics (40 nM) for 24 hrs, followed by BCG (MOI = 1) treatment for 48 hrs. The intracellular BCG in PBMac were obtained by lyzing the cells with 0.5% Triton-X 100 in PBS. The mycobacteria were plated on Middlebrook 7H10 agar plate and colonies formed were counted as CFU for quantification of BCG. The data are expressed as the mean ±SEM of independent experiments using PBMac from five individuals. n.s., not significant.
(PDF)

S4 Fig miR-1303 regulates TNF-α production. PBMac were transfected with miR-1303 mimics (40 nM) or negative control mimics (40 nM), followed by BCG (MOI = 1) treatment for 24 hrs. Cell supernatants were collected and the cytokine levels in the supernatants were determined by ELISA. The cytokine levels in cells were expressed as percentage relative to those in negative control mimics-BCG-treated cells. The data are expressed as the mean ±SEM of independent experiments using PBMac from six individuals. ***, p<0.001.
(PDF)
