# Supplementary figures and images for: MiR-1303 Regulates Mycobacteria Induced Autophagy by Targeting Atg2B
Source: PLoS One. 2016 Jan 15;11(1):e0146770. doi: 10.1371/journal.pone.0146770 (PMC4714759; doi:10.1371/journal.pone.0146770)

**S1 Fig.**

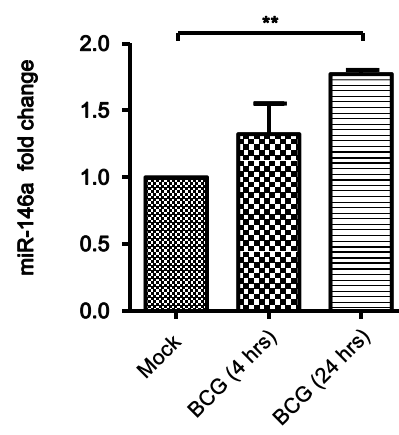

Supplement: S1 Fig — PBMacs were treated with Mock (diluent for BCG) for 4 hrs and 24 hrs respectively and BCG (MOI = 1) for indicated intervals. Total RNA was extracted and reverse-transcribed into cDNA. The miR-146a levels normalized to that of RNU48 were analyzed by quantitative PCR using the cDNA. The miR-146a levels in cells treated with BCG were expressed as fold change relative to those in mock-treated cells respectively. The data are expressed as the mean ±SEM of independent experiments using PBMacs from three individuals. **, p<0.01. (PDF) [file pone.0146770.s001.pdf]

**S2 Fig.**

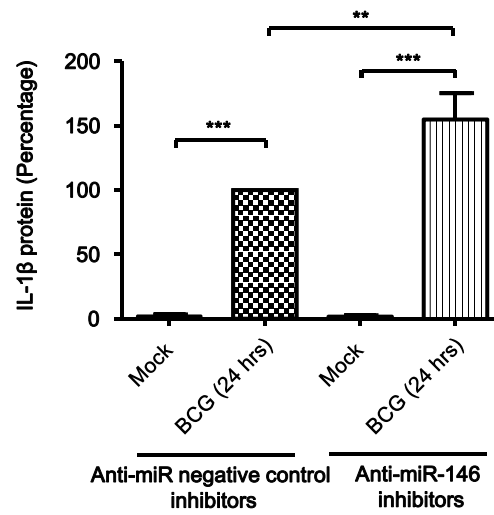

Supplement: S2 Fig — PBMac were transfected with Anti-miR Negative control inhibitors (40 nM) or Anti-miR-146a inhibitors (40 nM) for 24 hrs, followed by BCG (MOI = 1) treatment for 24 hrs. Cell supernatants were collected and the cytokine levels in the supernatants were determined by ELISA. The cytokine levels in cells were expressed as percentage relative to those in Anti-miR Negative control inhibitor-BCG (24 hrs)-treated cells. The data are expressed as the mean ±SEM of independent experiments using PBMac from seven individuals. **, p<0.01. ***, p<0.001. (PDF) [file pone.0146770.s002.pdf]

S3 Fig.

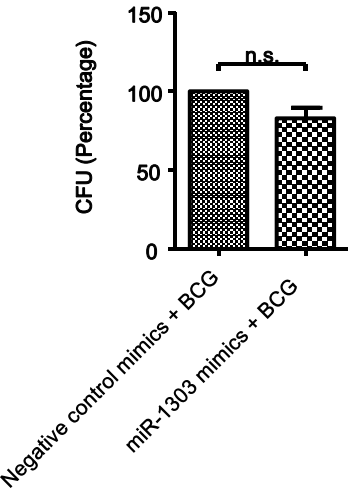

Supplement: S3 Fig — PBMacs were transfected with miR-1303 mimics (40 nM) or negative control mimics (40 nM) for 24 hrs, followed by BCG (MOI = 1) treatment for 48 hrs. The intracellular BCG in PBMac were obtained by lyzing the cells with 0.5% Triton-X 100 in PBS. The mycobacteria were plated on Middlebrook 7H10 agar plate and colonies formed were counted as CFU for quantification of BCG. The data are expressed as the mean ±SEM of independent experiments using PBMac from five individuals. n.s., not significant. (PDF) [file pone.0146770.s003.pdf]

S4 Fig.

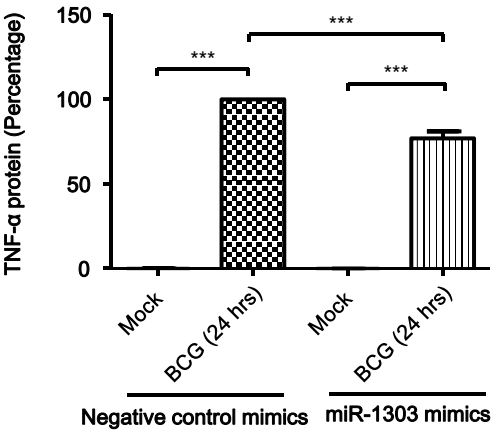

Supplement: S4 Fig — PBMac were transfected with miR-1303 mimics (40 nM) or negative control mimics (40 nM), followed by BCG (MOI = 1) treatment for 24 hrs. Cell supernatants were collected and the cytokine levels in the supernatants were determined by ELISA. The cytokine levels in cells were expressed as percentage relative to those in negative control mimics-BCG-treated cells. The data are expressed as the mean ±SEM of independent experiments using PBMac from six individuals. ***, p<0.001. (PDF) [file pone.0146770.s004.pdf]
